# Artificial intelligence-enabled cross-scale integration of traditional Chinese medicine and biomedicine for sepsis: from mechanisms to delivery

**DOI:** 10.3389/fphar.2026.1879407

**Published:** 2026-09-08

**Authors:** Ying-ying Cai, Yi Luo, Jie Yang

**Affiliations:** 1 Guangxi University of Chinese Medicine, Nanning, China; 2 Liuzhou Traditional Chinese Medicine Hospital Affiliated to Guangxi University of Chinese Medicine, Liuzhou, China; 3 Department of Emergency, Liuzhou Traditional Chinese Medicine Hospital, Liuzhou Integrated Chinese and Western Medicine Treatment Center for Snakebite, Liuzhou, China; 4 Clinical Research Center, The Second Affiliated Hospital of Guangxi Medical University, Nanning, China

**Keywords:** artificial intelligence, Chinese medicine and biomedicine, cross-scale integration, microenvironment responsiveness, molecular targets, nanodelivery systems, precision subtyping, sepsis

## Abstract

Sepsis is an organ dysfunction caused by a dysregulated host immune response to infection. Its pronounced heterogeneity and cross-scale pathological disturbances have resulted in poorly defined core therapeutic targets and inefficient drug delivery. Artificial intelligence, with its capacity for high-dimensional data integration, can serve as a data-integrative and hypothesis-generating tool, offering new avenues for exploring potential solutions to the aforementioned bottlenecks. This review summarizes recent advances in AI-driven multi-omics-based mechanistic dissection, the use of nanodelivery systems to optimize the *in vivo* behavior of both biomedical and botanical drugs, and AI-assisted nanocarrier design. At the mechanistic level, AI integrates multi-omics data with graph neural networks to provide computational clues for precise molecular subtyping and the identification of potential candidate targets such as S100A8/A9 and TREM-1. At the delivery level, nanocarriers help overcome the off-target toxicity of biomedical agents and the poor bioavailability of botanical drugs, while lesion acidification, high ROS levels, high MMP expression, and the EPR effect provide a biological basis for stimuli-responsive delivery. At the integration level, AI translates target information and microenvironmental parameters into carrier design parameters, offering computational support for material screening and response threshold optimization. A conceptual framework for an integrated “target recognition–drug matching–carrier design–subtype adaptation” decision model is proposed, which may inform the matching of combined biomedical and botanical drug regimens with nanodelivery systems based on patient molecular subtypes. This cross-scale integration framework may offer a reference direction for research on sepsis and other heterogeneous inflammatory diseases.

## Graphical Abstract

**Figure:**
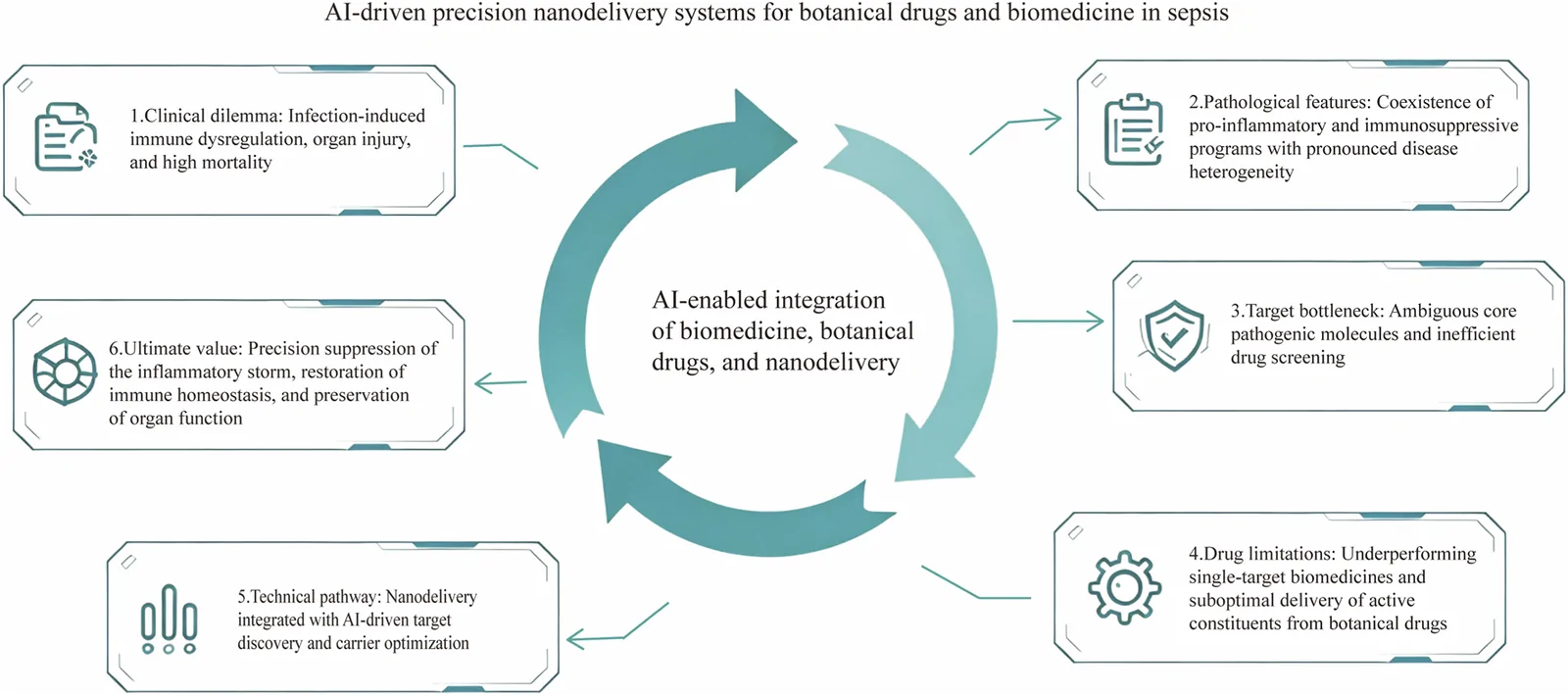

## 1 Introduction

Sepsis is an organ dysfunction syndrome triggered by a dysregulated host immune response to infection and remains a highly lethal condition in critical care medicine. It accounts for approximately 50 million incident cases and 11 million attributable deaths annually, representing nearly 20% of global mortality (Meyer and Prescott, 2024). Its profound heterogeneity, dynamic evolution, and cross-scale pathological dysregulation pose formidable challenges to precision therapy (Giamarellos-Bourboulis et al., 2024; Kwok et al., 2023). Despite continuous advances in treatment, transformative therapies targeting core pathophysiological processes remain exceedingly limited. The traditional paradigm of an early pro-inflammatory phase followed by late immunosuppression has been revised; contemporary single-cell and multi-omics studies demonstrate that pro-inflammatory and immunosuppressive programs coexist and intertwine from the earliest stages of disease, and that their dynamic interplay determines disease trajectory and the extent of organ injury (Giamarellos-Bourboulis et al., 2024; Kwok et al., 2023). The intricate pathological networks of sepsis directly obscure core pathogenic molecular targets, leaving a lack of unified and definitive molecular rationale for intervention. Concurrently, conventional reductionist and empirical research paradigms, characterized by low efficiency, are unable to effectively identify therapeutic molecules and targeting biomarkers aligned with the pathological features of sepsis. The chronic absence of high-throughput, high-precision screening platforms has ultimately resulted in a low translational success rate for targeted drug development and has hindered the clinical implementation of precision diagnosis and treatment.

The ambiguity of molecular targets and the lack of robust screening technologies have directly contributed to the current drug development impasse for both biomedicine and botanical drugs in sepsis. Biomedicines offer a distinct advantage in rapidly controlling inflammation through single-target intervention; however, their development is constrained by lengthy timelines, prohibitive costs, low success rates, and, crucially, the inability of single-target strategies to contend with the complex, networked pathological mechanisms of sepsis (Marissa et al., 2023). Phase III trials of tumor necrosis factor-α (TNF-α) antagonists and interleukin-1 (IL-1) receptor antagonists have repeatedly failed, confirming that blockade of a single target cannot halt the inflammatory storm mediated by multiple redundant pathways in sepsis. In contrast, active constituents of botanical drugs possess the unique advantage of multi-pathway, multi-target immunomodulation, which is well-suited to the heterogeneous pathology of sepsis; yet these compounds are commonly plagued by poor aqueous solubility, inadequate *in vivo* stability, and insufficient targeting to disease foci, severely limiting their clinical translation (Baidoo et al., 2025).

The advent of intelligent nanodelivery materials has emerged as a pivotal carrier to compensate for the delivery shortcomings of both biomedicine and botanical drugs, thereby enabling precision therapy for sepsis. To address the high off-target rate and severe systemic toxicity of single-target biomedicines, nanocarriers can achieve site-specific drug enrichment at disease foci through controlled loading and targeted modification, reducing non-specific exposure in normal tissues and improving both therapeutic safety and efficacy (Brusini et al., 2020). To overcome the poor aqueous solubility, rapid metabolic clearance, and low bioavailability of active constituents in botanical drugs, nano-encapsulation technology can effectively improve their physicochemical properties, prolong systemic circulation time, resist enzymatic degradation *in vivo*, and fully preserve the multi-component, multi-pathway regulatory advantages of botanical drugs (Garg et al., 2015). Furthermore, the septic inflammatory microenvironment possesses distinctive pathological features—local acidosis, excessive generation of reactive oxygen species (ROS) and reactive nitrogen species (RNS), overexpression of inflammation-associated enzymes such as matrix metalloproteinases (MMPs), and a compromised vascular endothelial integrity coupled with impaired local lymphatic drainage, which collectively mediate the enhanced permeability and retention (EPR) effect. This provides a natural biological foundation for targeted delivery and controlled release by microenvironment-responsive nanodelivery systems (Ismail et al., 2022).

Although nanomaterials can provide delivery support for anti-sepsis molecules from both biomedical and botanical sources, current nanocarrier design remains largely dependent on empirical trial-and-error, with common issues including unclear targeting addresses, unsubstantiated response parameters, and homogenized carrier design. The root cause of these problems lies in the lack of systematic approaches for mining target information and lesion-specific signaling features from patient multi-omics data. Artificial intelligence (AI) refers to a technological system that simulates human intelligence through algorithms and computational models, encompassing in the biomedical field machine learning (supervised and unsupervised learning), deep learning (CNN, RNN/LSTM, GNN, Transformer), natural language processing, reinforcement learning, generative models, and federated learning, among other branches (Topol, 2019). Compared with traditional trial-and-error R&D paradigms, AI can serve as a data-driven analytical tool, providing hypothetical directions and design references for addressing the two major bottlenecks of unclear molecular targets and lack of quantitative basis for nanocarrier design, although it cannot independently establish biological causality. AI can also attempt to integrate multi-omics molecular profiles with multimodal clinical data to generate testable research hypotheses for sepsis molecular target discovery, nanocarrier optimization, and biomedical and botanical drug delivery research. In the area of molecular target discovery, AI can assist in high-throughput screening of sepsis-associated candidate targets from massive complex biological data, providing direction for further mechanistic studies (Vamathevan et al., 2019). In nanodelivery and drug development, AI can attempt to extract and quantify microenvironmental signaling features and cell-targeting biomarkers of septic lesions, generating experimentally testable hypotheses for the targeted modification, responsive mechanism design, and release parameter optimization of smart nanocarriers.

Nanodelivery technology can help overcome the delivery deficiencies of biomedical and botanical drugs. However, the development of traditional nanocarriers lacks precise targets as design guidance; AI, with its capacity for multidimensional biological data mining, can fill these two gaps—missing targets and the lack of quantitative basis for nanocarrier design—thereby forming a complete research chain of AI-assisted target discovery, nanodelivery-enabled therapy, and integrated biomedical-botanical treatment. In summary, AI can bidirectionally link molecular target mining and smart nanodelivery optimization, offering new approaches for bridging the technological gaps among sepsis pathophysiological research, biomedical and botanical drug development, and precision delivery, while providing a conceptual framework for constructing an intelligent and precise nanoplatform for integrated biomedical and botanical drug delivery. Through AI-assisted screening of candidate pathogenic targets in sepsis, attempts to quantify lesion-targeting features, and computational optimization of nanodelivery system parameters, testable hypotheses can be generated for laboratory-level exploration of targeted interventions against sepsis-associated molecular pathways using biomedical and botanical agents. Substantial experimental validation remains necessary before such approaches can yield effective strategies for alleviating the systemic inflammatory response and multi-organ damage in sepsis.

## 2 AI-driven multi-omics and multimodal data analysis for deciphering septic pathogenesis and molecular targets

### 2.1 Pathological complexity of sepsis and inherent limitations of traditional mechanistic research and target screening

The core pathological mechanism of sepsis is not a linear dysregulation of a single molecular pathway, but rather a cross-scale immune disorder involving multiple organs, multiple cell types, and interwoven signaling networks throughout the body (Singer et al., 2016). In the early stage of the disease, excessive inflammatory activation and immunosuppressive programs can coexist, intertwining and evolving dynamically, resulting in a highly individualized and temporally variable disease trajectory. This distinctive pathological feature directly leads to difficulties in precisely elucidating the core pathogenic mechanisms of sepsis, with key interventional molecular targets remaining unclear, representing a critical upstream bottleneck constraining the development of precision diagnostics and therapy.

For a long time, sepsis mechanistic research and target discovery have relied heavily on traditional biological experiments and single-dimensional approaches, which carry significant technical limitations. Traditional studies, predominantly centered on single-omics profiling and stepwise single-pathway validation, have struggled to systematically capture the cascading regulatory relationships among genes, proteins, immune cells, and the tissue microenvironment, thereby failing to precisely dissect the global, networked immune dysregulation mechanisms underlying sepsis (Meyer and Prescott, 2024). Conventional target screening, largely based on empirical selection and low-throughput experimental validation, suffers from low efficiency and strong subjective bias, making it difficult to identify from the vast array of biomolecules the core pathogenic hub molecules that truly drive disease progression and mediate organ injury. This has ultimately resulted in the failure of numerous candidate targets during clinical validation (Vamathevan et al., 2019; Wouters et al., 2020). Sepsis patients exhibit significant interindividual heterogeneity, with those differing in infection type, disease stage, and immune status presenting entirely distinct pathological phenotypes. Traditional research, however, lacks precise disease subtyping criteria, and the homogenized, one-size-fits-all research and therapeutic approaches fail to accommodate individualized pathological features—a major reason for the variability in clinical outcomes and the hindered implementation of precision diagnostics and therapy.

### 2.2 AI integration of multi-omics data to deconstruct the cross-scale pathogenic regulatory network in sepsis

In response to the aforementioned bottlenecks, AI, with its capacity for high-dimensional data integration and nonlinear association mining, offers new analytical tools for exploring viable pathways to systematically deconvolute the cross-scale pathogenic regulatory networks of sepsis. At the level of multi-omics data integration, machine learning and deep learning algorithms can integrate genomic, transcriptomic, proteomic, metabolomic, and single-cell omics data into a unified analytical framework for combined analysis. Taking transcriptomic analysis as an example, AI can assist in performing unsupervised clustering analysis on large-scale RNA sequencing data from peripheral blood mononuclear cells of sepsis patients, helping to identify gene co-expression modules associated with disease severity and clinical prognosis—most of which correspond to characteristic immunopathological processes such as neutrophil degranulation, T-cell exhaustion, and mitochondrial oxidative phosphorylation dysfunction (Timothy et al., 2015; Davenport et al., 2016). Building on this, AI can longitudinally link transcriptomic, proteomic, and metabolomic data, providing clues for reconstructing the molecular cascade of gene expression dysregulation, protein abnormalities, and metabolic homeostasis imbalance, while hinting at potential pathogenic associations that traditional studies may overlook. At the level of regulatory network analysis, graph neural networks (GNNs) enable structured modeling and deep inference of complex biological networks. By setting genes, proteins, and metabolites as network nodes, and protein-protein interactions, transcriptional regulation, and signal transduction relationships as network edges, sepsis-specific gene-protein-immunity pathway interaction networks can be constructed (Ga et al., 2021). GNNs can learn node feature representations within heterogeneous biological networks, aiding in the prediction of potential hub molecules that may occupy topologically central positions within the network. Multiple studies have employed GNNs to construct sepsis interaction networks, predicting potential hub molecules such as NF-κB and TLR4 (Meyer and Prescott, 2024). It is important to emphasize that the biological functions of the aforementioned molecules were all subsequently confirmed through wet-lab experiments; network analysis alone cannot establish causal relationships. Subsequent functional validation further confirmed that these molecules serve both as core signaling nodes for pro-inflammatory signals and as participants in NLRP3 inflammasome activation-induced pyroptosis and the transcriptional regulation of immunosuppressive factors such as IL-10. These findings provide a preliminary network analytical framework for understanding the intertwined regulatory patterns of three major pathological programs—hyperinflammation, immunoparalysis, and pyroptosis—during the course of sepsis, representing a systemic-level insight that traditional reductionist single-pathway approaches have struggled to achieve.

Furthermore, AI-driven high-dimensional feature mining holds promise for assisting in distinguishing protective inflammatory signals from pathogenic inflammatory molecular signatures. However, this can only generate hypotheses based on data correlations, with final conclusions depending on *in vivo* and *in vitro* functional validation. This direction carries significant reference value for the design of sepsis therapeutic strategies: indiscriminate suppression of inflammatory responses can readily lead to the collapse of host immune defense. Through comparative learning using omics data from sepsis patients with different prognoses, AI can attempt to identify pathogenic inflammatory molecular features that are exclusively associated with poor prognosis and organ failure, without participating in pathogen clearance. These findings not only advance the theoretical understanding of the dynamic molecular mechanisms underlying sepsis progression but also provide a theoretical foundation for the development of precision intervention strategies that target pathogenic inflammation while preserving normal host immune defense.

### 2.3 AI-driven multimodal data fusion enables precision subtyping and pathological characterization of sepsis

The pronounced heterogeneity of sepsis dictates that “molecular mechanistic dissection” must ultimately serve “precision patient subtyping.” AI’s advantages in multimodal data fusion provide important technical tools for addressing the challenge of clinical heterogeneity in sepsis. Multimodal data fusion refers to AI’s ability to integrate data types from entirely different sources—including but not limited to molecular profiling data such as transcriptomics and proteomics, as well as time-series vital signs, routine clinical laboratory parameters, medical imaging, and electronic health record text—into a unified deep learning architecture for joint modeling. For example, a typical multimodal model can simultaneously receive continuous heart rate, blood pressure, lactate levels, C-reactive protein, and procalcitonin levels recorded within 72 h of ICU admission, along with peripheral blood single-cell transcriptomic data obtained on the day of admission. Through attention mechanisms, the model automatically learns cross-modal correlation weights, ultimately generating a comprehensive assessment of the patient’s current immune status. Studies by Acosta and colleagues have demonstrated that such multimodal fusion frameworks can significantly improve the prediction accuracy of adverse outcomes in intensive care settings (Acosta et al., 2022).

In precision subtyping applications, these AI models have shown considerable potential. Sweeney et al. (Timothy et al., 2015), through unsupervised clustering analysis of peripheral blood transcriptomic data from sepsis patients, first identified several clinically distinct subtypes with significant molecular differences, among which the most critical subtyping dimension was the distinction between “pro-inflammatory dominant/hyperinflammatory” and “immunosuppressive dominant/immunoparalytic” phenotypes. Davenport et al. (Davenport et al., 2016) further validated this subtyping framework and confirmed that different transcriptomic subtypes are significantly associated with clinical prognosis, with immunoparalytic patients exhibiting higher long-term mortality. Scicluna et al. (Scicluna et al., 2017) similarly identified analogous molecular subtyping patterns in cohorts of severe infection, underscoring the importance of host response heterogeneity in therapeutic decision-making. These two core subtypes differ not only in transcriptomic profiles—hyperinflammatory subtype characterized by high expression of NF-κB target genes and inflammatory cytokine storm genes, immunoparalytic subtype by downregulation of HLA-DR, upregulation of PD-L1, and T-cell exhaustion markers—but also exhibit significant differences in clinical presentation and prognosis: hyperinflammatory patients experience more prominent early circulatory failure, while immunoparalytic patients face higher risks of secondary infections and delayed deterioration with multi-organ failure. Seymour et al. (Seymour et al., 2019), based on large multicenter cohort clinical data, similarly confirmed the existence of reproducible clinical phenotype classifications in sepsis, with different phenotypes showing markedly divergent responses to therapeutic interventions. An important value of AI-based subtyping lies in its ability to stratify sepsis patients who present with seemingly similar clinical manifestations into subgroups with fundamentally distinct molecular mechanisms, thereby providing a foundation for subsequent differentiated targeted therapies.

AI can also attempt to reveal intrinsic molecular differences between patients with different disease courses and prognoses, elucidating the differential mechanisms underlying organ injury. By incorporating time-series vital signs and imaging data—such as quantitative CT analysis of pulmonary edema and bedside ultrasound assessment of renal blood perfusion—into multimodal models, AI can facilitate the establishment of cross-scale mapping frameworks connecting “molecular pathway aberrations, specific organ microenvironmental alterations, and imaging/clinical phenotypes of organ dysfunction.” For instance, models may reveal that acute kidney injury in a particular subtype is primarily associated with mitochondrial dysfunction and excessive activation of oxidative stress signaling, rather than simple renal hypoperfusion—a pathogenic mechanism that has been confirmed by multiple experimental studies. AI-based multimodal integrative analysis can provide reference pathways for translating such mechanistic insights into actionable individual subtyping decisions (Hernando et al., 2014). The value of AI-powered multimodal subtyping extends beyond classification itself, lying in its capacity to convert clinical heterogeneity into measurable molecular features amenable to further investigation.

### 2.4 High-throughput screening of core pathogenic molecular targets in sepsis based on AI algorithms

The ultimate goal of elucidating mechanisms and achieving precise subtyping is to identify actionable core pathogenic molecular targets. This represents the upstream core innovation of the entire logical chain and serves as the prerequisite and foundation for subsequent drug screening and nanocarrier-based targeted delivery. AI has provided new perspectives for transitioning target discovery from the traditional “low-throughput hypothesis-driven” paradigm toward a “high-throughput data-driven” approach, with core technical pathways primarily encompassing differential molecule mining, network key node identification, and multi-omics evidence integration (Pun et al., 2023). At the differential molecule mining level, feature selection algorithms such as random forests, gradient boosting trees, and deep neural networks can score and rank tens of thousands of genes or proteins based on their expression differences, prognostic relevance, and clinical associations, generating unbiased candidate target lists at the genome-wide level (Pun et al., 2023). At the network key node identification level, AI integrates network analysis methods with graph neural networks to generate hypothetical maps that project the aforementioned differential molecules onto protein-protein interaction networks and signaling pathway databases (Ga et al., 2021). Through message-passing mechanisms, GNN models learn the topological structure and information flow of the entire network, enabling precise computation of topological importance metrics such as “centrality,” “betweenness,” and “bottleneck” for each node within the network (Zhang et al., 2021). Based on these topological features, AI can screen for molecules with potential as pathogenic driver nodes (candidate targets). However, computational analysis alone cannot strictly distinguish between true driver nodes that actively regulate disease progression and passive responder nodes that merely serve as downstream biomarker signatures; functional experiments are indispensable for making this distinction (Nogales et al., 2022). Using this approach, multiple independent studies have screened and validated molecules including S100A8/A9 heterodimer, TREM-1, PD-L1, and STING as core pathogenic hubs in sepsis, with gene knockout or pharmacological blockade significantly improving organ injury and survival in animal models (Dave et al., 2025; François et al., 2023).

Notably, for specific organ dysfunctions such as sepsis-associated lung injury, acute kidney injury, and liver injury, AI can assist in screening organ-specific candidate targeting molecules. The underlying principle involves integrating single-cell omics data from specific organ tissues (e.g., lung tissue, renal tubular epithelial cells) with organ injury-related gene signatures from public datasets, employing contrastive learning algorithms to identify molecules that are highly expressed in specific organs and contribute most significantly to organ injury under conditions of systemic immune dysregulation (Mogilenko et al., 2021; Theodoris et al., 2023). The discovery of these organ-specific targets provides precise “targeting addresses” for the design of nanodelivery systems that are “both mechanistically targeted and enriched at injured organs.” For instance, in sepsis-associated acute lung injury, AI analysis can identify adhesion molecules or scavenger receptors specifically upregulated on pulmonary vascular endothelial cells or alveolar macrophages as targets; for sepsis-associated acute kidney injury, novel surface antigens exposed during renal tubular epithelial cell stress can be identified (Grommes and Soehnlein, 2011; Mason et al., 2016). It should be emphasized that AI-based target screening generates hypotheses based on data correlations and cannot directly establish causal pathogenic mechanisms. All candidate targets must undergo *in vivo* and *in vitro* functional validation before proceeding to drug screening and nanocarrier design stages.

## 3 Nanodelivery systems overcome the delivery bottlenecks of anti-sepsis biomedicines and botanical drugs and their microenvironment-responsive foundation

Integrating the findings from AI-driven multi-omics mechanistic dissection, multimodal precision subtyping, and high-throughput target screening, the pathogenic targets that have been experimentally validated or supported by clear literature provide a theoretical foundation for targeted therapy in sepsis. However, free drugs administered systemically face significant delivery bottlenecks, as they cannot achieve precise accumulation at inflammatory lesions *in vivo* (Peer et al., 2007). This creates a translational challenge for anti-sepsis biomedical and botanical drugs—where “targets are actionable, yet drugs fail to take effect.” Unlike conventional active pharmaceutical ingredients (APIs), nanomaterials are characterized by their “delivery carrier” property rather than “therapeutic activity” *per se*. Conventional small-molecule drugs, following systemic administration, distribute uniformly throughout the body, lacking lesion-targeting capacity and being subject to rapid clearance. In contrast, nanomaterials can evade renal filtration through size control (10–200 nm), reduce reticuloendothelial system clearance via PEGylation, and passively accumulate at inflammatory lesions through the EPR effect, while further enabling active targeting recognition through surface ligand modification. More importantly, nanocarriers can co-load biomedical and botanical drugs with vastly different physicochemical properties within a single system, enabling multi-drug synergistic delivery. Simultaneously, leveraging the unique microenvironmental signals of septic lesions—acidification, high ROS, and high MMP levels—responsive release can be engineered to ensure that drugs are “released at the right time and the right location.” Therefore, the relevance of nanomaterials in this review lies in their role as the physical bridge connecting “target discovery” with “precision intervention”—a critical technological vehicle for overcoming drug delivery bottlenecks, rather than an independent therapeutic agent. At the laboratory research level, nanodelivery systems, with their physicochemical tunability, lesion-targeting accumulation capacity, and controlled release characteristics, offer conceptual and technical references for exploring feasible strategies to overcome the delivery shortcomings of biomedical and botanical drugs, with the potential to synergistically harness the rapid anti-inflammatory effects of biomedical agents and the multi-pathway immunomodulatory advantages of botanical drugs (Sun et al., 2025).

### 3.1 Nanocarrier-optimized anti-inflammatory biomedicines

Anti-inflammatory biomedicines for sepsis treatment are characterized by “well-defined targets but uncontrolled delivery.” Single-target biomedicines, including glucocorticoids, TNF-α antagonists, IL-1 receptor antagonists, and TLR4 and NF-κB pathway inhibitors, can precisely intercept inflammatory cascades and rapidly suppress the inflammatory storm, rendering them suitable for early emergency intervention in sepsis (Karakike and Giamarellos-Bourboulis, 2019). However, free drugs lack lesion-targeting capability and cannot distinguish pathogenic inflammation from protective host immunity, readily causing systemic adverse effects—a core reason for the disappointing efficacy of most anti-inflammatory drugs in phase III clinical trials (Tom et al., 2021). Following systemic infusion, free small-molecule anti-inflammatory drugs distribute uniformly throughout bodily tissues, with only a small fraction accumulating in inflamed and injured organs such as the lungs and kidneys, while the majority of the drug remains in normal tissues, eliciting pronounced off-target effects (Peer et al., 2007). Sepsis is characterized by the coexistence of both excessive inflammatory activation and immunosuppression; broad-spectrum anti-inflammatory intervention non-selectively suppresses host antimicrobial immunity, exacerbates T-cell exhaustion and immune paralysis, and substantially elevates the risks of secondary infection and delayed-onset multi-organ failure (Torres et al., 2022). Free drugs are susceptible to degradation by plasma enzymes and undergo rapid hepatic and renal clearance, resulting in short circulating half-lives that necessitate high-dose, high-frequency administration. Long-term intervention readily causes hepatic and renal function impairment, endocrine disorders, and other toxic side effects, severely compromising clinical medication safety (Sun et al., 2025).

Nanocarriers can optimize the *in vivo* behavior of biomedical agents across multiple dimensions—including circulatory stability, lesion-targeting capability, and safety profile—offering a viable pathway for reducing toxicity while enhancing efficacy (Peng et al., 2025). Encapsulating or conjugating drugs within nanocarriers with controlled particle sizes ranging from 50 to 200 nm can circumvent rapid renal filtration and prolong circulation time; surface PEGylation reduces clearance by the reticuloendothelial system, thereby improving blood stability. Leveraging the EPR effect resulting from the hyperpermeability of vasculature at septic lesions, nanomedicines can achieve specific accumulation at injured tissues, enabling localized and precise blockade of pathogenic inflammatory pathways while largely preserving normal immune defense. Numerous animal studies have demonstrated that anti-inflammatory agents loaded in liposomal or PLGA carriers can significantly increase drug concentrations in pulmonary lesions while reducing the risk of systemic immunosuppression. This spatially confined nanocarrier-based intervention approach thus offers a feasible strategy for mitigating the central challenge in sepsis therapy—the inherent conflict between “achieving potent anti-inflammatory effects” and “avoiding excessive immunosuppression.”

### 3.2 Nanocarrier-optimized active constituents of botanical drugs

Compared with the single-pathway blockade mode of biomedicines, active constituents of botanical drugs possess the unique advantage of multi-pathway, bidirectional immunomodulation, rendering them better suited to the complex, networked immune dysregulation mechanisms of sepsis. Representative active monomers such as tanshinone IIA, baicalein, and emodin can simultaneously inhibit NF-κB-mediated inflammatory storms, scavenge excess reactive oxygen species (ROS) at disease foci, and block NLRP3 inflammasome activation, while also improving T-cell function and partially reversing the immunosuppressive state, thereby achieving the ideal therapeutic outcome of “suppressing inflammation without compromising immunity” (Zhong‐ying et al., 2021). However, these compounds are predominantly lipid-soluble monomers and share common intrinsic deficiencies, including poor aqueous solubility, limited *in vivo* stability, and exceedingly low bioavailability. Following administration in free form, they are prone to hydrophobic precipitation in the bloodstream or rapid enzymatic degradation and inactivation, exhibiting short half-lives. Coupled with a pronounced hepatic first-pass effect, drug concentrations at disease foci rarely attain effective therapeutic thresholds, preventing the full realization of their multi-target regulatory advantages.

Nanopackaging technologies provide a technical means to address the aforementioned delivery challenges without altering the multi-target pharmacological nature of botanical active ingredients. By constructing core-shell structures such as micelles, liposomes, and PLGA nanoparticles, the hydrophobic cores can efficiently encapsulate lipophilic compounds, significantly improving water solubility and blood stability while enabling safe administration. The physical barrier formed by the carrier shell protects against enzymatic and acid-base degradation, prolongs circulation time, and partially bypasses hepatic first-pass metabolism through lymphatic transport pathways, substantially enhancing bioavailability (Qiu et al., 2023). Tanshinone IIA serves as an illustrative example: multiple studies have demonstrated that its oral bioavailability following nanoformulation is increased several-fold to tens of fold compared with the free drug. In the cecal ligation and puncture (CLP) sepsis model, nanoformulated tanshinone IIA significantly downregulated TNF-α and IL-6 levels, while ameliorating pulmonary edema and oxidative inflammatory injury, thereby providing a candidate delivery platform for botanical monomer-based intervention in sepsis (Sun et al., 2025; Lin et al., 2026).

### 3.3 Foundation for constructing inflammation microenvironment-responsive nanodelivery systems

Passive targeting strategies based on the EPR effect are constrained by interpatient pathological variability, non-specific adsorption, and premature drug leakage, resulting in limited lesion accumulation efficiency and uncontrollable release kinetics that fail to match the dynamically evolving pathological features of sepsis (Yang et al., 2026). The unique biochemical microenvironment of septic inflammatory lesions provides precise endogenous triggers for microenvironment-responsive nanodelivery systems. Integrating multiple microenvironment-responsive mechanisms aims to achieve spatiotemporally controlled delivery characterized by “stable sequestration in circulation, site-specific drug release at lesions, and deep tissue penetration,” representing a core strategy for enhancing the targeting precision and therapeutic safety of both biomedical and botanical drugs (Yang et al., 2026).

The inflammatory storm in sepsis induces the formation of four characteristic pathological microenvironmental features in target organs such as the lungs and kidneys: lesion acidification, excessive ROS accumulation, aberrant overexpression of MMP-2/MMP-9, and EPR effects mediated by increased vascular permeability (Hossan et al., 2025). These four features synergistically provide a dual pathological basis for passive accumulation and controlled drug release of nanocarriers. The EPR effect serves as the structural prerequisite for passive targeting of nanomedicines. The intact endothelial barrier in normal vasculature blocks non-specific nanoparticle extravasation; in contrast, inflammatory injury in sepsis compromises endothelial integrity and widens vascular gaps, allowing appropriately sized nanoparticles to extravasate through damaged vessels and specifically accumulate at lesions, thereby achieving spatial distinction between diseased and normal tissues (Chen et al., 2023). Lesion acidification constitutes the core trigger for pH-responsive drug release. While normal tissues maintain a pH of approximately 7.4, infiltrating immune cells in sepsis undergo glycolytic metabolic reprogramming, generating large amounts of lactic acid and other acidic products that reduce the extracellular pH of lesions to 6.0–6.8. Leveraging this differential, nanocarriers incorporating acid-labile structures such as acetal, hydrazone, and Schiff base linkages can stably retain drugs in neutral blood while specifically cleaving and releasing drugs in acidic lesions, effectively avoiding off-target release in normal tissues (Dai and Sun, 2026). The high-ROS microenvironment of lesions provides pathological support for oxidation-responsive delivery. Activated neutrophils and macrophages trigger oxidative bursts through NADPH oxidase, resulting in ROS concentrations far exceeding normal levels. Excessive ROS serves both as a core pathogenic factor mediating organ cell injury and as a specific hallmark of inflammatory lesions. Nanocarriers incorporating ROS-sensitive moieties such as thioether, thioketal, and phenylboronic ester groups can undergo specific degradation and drug release in high-ROS environments, with degradation products synergistically scavenging excess ROS to achieve dual benefits of targeted release and microenvironmental repair. High MMP expression at lesions provides the biological basis for enzyme-responsive delivery. MMP-2 and MMP-9 are expressed at low levels in normal tissues but are aberrantly overexpressed in septic inflammatory regions, where they degrade the extracellular matrix and exacerbate inflammatory infiltration and organ injury. By modifying the carrier surface with MMP-specific substrate peptides, the highly expressed MMPs at lesions can precisely cleave functional peptide segments, triggering carrier dissociation and site-specific drug release—thereby achieving responsive, controllable delivery based on lesion-specific biological characteristics (Yang et al., 2026). The synergistic action of multiple microenvironment-responsive mechanisms helps overcome the inherent limitations of conventional passive targeting, particularly its lack of spatiotemporal control over drug release.

In summary, microenvironment-responsive nanodelivery systems offer a technical strategy for alleviating the challenges of inefficient delivery and severe off-target injury associated with biomedical and botanical drugs *in vivo*. However, current structural design and targeting modification strategies for nanocarriers remain largely dependent on empirical *in vitro* trial-and-error screening, lacking precise lesion target information and quantified microenvironmental parameters as design foundations. The prevalence of homogenized carrier design hinders the achievement of individualized targeted delivery tailored to different sepsis subtypes.

## 4 AI-driven rational design of intelligent nanodelivery systems and integration of biomedicine and botanical drugs

### 4.1 From empirical trial-and-error to rational design: a paradigm shift in nanocarrier development

AI integration of multi-omics and multimodal data provides a foundation for constructing pathogenic molecular target libraries and organ-specific targeting biomarkers. The value of nanodelivery systems in optimizing biomedical and botanical drugs, along with the responsiveness to four types of inflammatory microenvironmental signals, has been established. However, current nanocarrier development remains heavily reliant on empirical trial-and-error, presenting several challenges: the selection of targeting ligands lacks single-cell-level precision molecular evidence, and organ-specific biomarkers identified by AI have not yet been translated into ligand design parameters; microenvironment-responsive thresholds are determined empirically, with critical values for lesion-triggered drug release lacking *in vivo* calibration; and large-scale carrier material screening remains inefficient, with widespread carrier structural homogenization (Bae et al., 2025). Leveraging its capacity for high-dimensional data fusion and nonlinear modeling, AI can integrate target information, quantified microenvironmental parameters, and nanodelivery mechanisms within a unified computational framework, offering technical potential for transitioning nanodelivery systems from traditional empirical trial-and-error paradigms toward data-supported design approaches (Bae et al., 2025).

### 4.2 AI-driven nanocarrier material screening and drug-carrier matching prediction

Traditional nanocarrier screening requires the sequential synthesis and characterization of hundreds of candidate materials, a process that is exceedingly time-consuming. AI can accomplish high-throughput virtual screening to identify candidate formulations within days (Yazdani et al., 2026). Machine learning algorithms, using carrier physicochemical parameters and the molecular features of both biomedical and botanical drugs as input variables, can rapidly predict critical formulation metrics such as drug loading capacity, encapsulation efficiency, and colloidal stability, thereby screening thousands of drug-carrier candidate combinations efficiently (Shen et al., 2026). Molecular dynamics simulations can reveal the microscopic behavior of drugs within carriers—for example, the aggregation state of tanshinone IIA in PLGA matrices, the depth of its incorporation into lipid bilayers, or its adsorption sites within the pores of ZIF-8 metal-organic frameworks—providing references for carrier chemical modification optimization (Alex and Tomasz, 2020). Previous studies have successfully employed machine learning to assist in screening liposome formulations, compressing the R&D timeline from months to 2 weeks. This methodological framework offers a valuable reference for nanocarrier screening in sepsis applications involving biomedical and botanical drugs (He et al., 2020).

### 4.3 AI-enabled precision targeting modification: molecular target-based ligand screening and surface optimization

Candidate targets identified through AI screening possess only statistical correlations and must undergo rigorous *in vivo* and *in vitro* functional validation—such as gene knockout and protein blockade studies—to establish causal relationships before they can be applied in the development of targeted therapeutics and carriers (Pun et al., 2023). The organ-specific targeting biomarkers identified through screening provide candidate “targeting addresses” for AI-guided ligand selection. Deep learning models can perform high-throughput prediction of binding affinities between candidate ligands (e.g., peptides, antibody fragments) and their targets, while generative adversarial networks can even *de novo* design novel targeting peptide sequences (Fangping et al., 2022). Furthermore, AI can evaluate the synergistic effects of dual-ligand co-modification, precluding unfavorable combinations such as steric hindrance or competitive binding between ligands. In optimizing surface modification parameters, AI can calculate optimal ligand density and PEG linker lengths to balance targeting efficiency with evasion of *in vivo* immune clearance (Gokay et al., 2019). Differentiated modification strategies can be implemented based on sepsis molecular subtyping results: for hyperinflammatory patients, prioritizing targeting of pulmonary ICAM-1 or scavenger receptors; for immunoparalytic patients, targeting T-cell PD-1 for co-delivery of immunorestorative agents—thereby providing references for differentiated targeting design (Timothy et al., 2015; Mason et al., 2016).

### 4.4 AI-optimized microenvironment-responsive parameters: from qualitative design to quantitative calibration

The qualitative understanding that “septic lesions are acidic” is far from sufficient to determine the precise drug release threshold of carriers; a substantial gap exists between qualitative understanding and quantitative design (Mitchell et al., 2021). The selection of release thresholds directly determines the efficiency of drug release at lesions and the risk of leakage into normal tissues, yet traditional empirical design has consistently lacked quantitative basis. AI can integrate measured microenvironmental parameters with the chemical properties of responsive materials to establish drug release kinetic models, using the “lesion drug exposure/normal tissue exposure” ratio as the optimization objective function to solve for optimal release trigger thresholds, thereby providing computational support for the transition from qualitative judgment to quantitative design (Wang et al., 2021). In ROS-responsive design, AI calculates the optimal molar ratios of sensitive moieties such as thioether bonds, while incorporating the ROS-scavenging capacity of carrier degradation products into synergistic optimization (Wang et al., 2021). In MMP-responsive design, AI customizes substrate peptide sequences with differential cleavage efficiencies for different lesions based on organ-specific protease expression profiles, and designs “pH + ROS” or “pH + MMP” multi-responsive logic gates to reduce drug mis-release triggered by single pathological signals, thereby enhancing delivery safety (Mura et al., 2013).

### 4.5 AI-integrated “target–drug–carrier–subtype” unified decision-making model

The ultimate therapeutic efficacy of nanodelivery systems is determined by multiple interrelated factors—including carrier materials, targeting ligands, responsive mechanisms, and encapsulated drugs—necessitating the construction of an end-to-end AI-integrated decision model as a research framework (Wang et al., 2021). The model input layer incorporates patient multimodal clinical data, encompassing molecular subtypes, target expression profiles, laboratory parameters, and imaging-based organ damage assessments. Following feature extraction through deep learning, the framework proceeds to three parallel modules: a drug-matching engine that integrates patient subtypes and pathogenic targets to recommend optimal combinations of biomedical and botanical drugs, while leveraging reinforcement learning algorithms to determine dosing timing and dosage; a carrier design engine that matches appropriate carrier materials and surface modification strategies based on drug physicochemical properties and the location of affected organs; and a delivery optimization engine that generates individualized dosing regimens through physiologically based pharmacokinetic (PBPK) modeling (Komorowski et al., 2018). As a theoretical illustrative example: for a hyperinflammatory patient with acute lung injury, an ICAM-1-targeted PLGA-PEG nanoparticle loaded with corticosteroids could be designed with pH 6.5-triggered rapid release; for an immunoparalytic patient, a ZIF-8 carrier co-loading tanshinone IIA and PD-1 inhibitors could be employed with MMP-responsive sustained release. This “one-patient, one-customized-nanocarrier” individualized model currently remains at the theoretical framework level, providing conceptual references for constructing research logic that integrates target resources, biomedical and botanical drugs, and nanodelivery systems through AI.

In summary, AI bidirectionally links upstream pathogenic target discovery with downstream nanodelivery system optimization, proposing the conceptual framework of a data-driven precision delivery research paradigm for sepsis. Leveraging multi-omics data to screen pathogenic targets and quantify lesion microenvironmental features on one end, while translating biological parameters into quantifiable metrics for carrier design on the other, this approach facilitates the transition of nanodelivery from empirical trial-and-error toward data-driven paradigms. Through this integrated platform, individualized matching strategies for biomedical and botanical drug combinations and nanodelivery regimens can be extrapolated based on patient molecular subtypes and organ injury characteristics, offering research perspectives and technical references for exploring the construction of spatiotemporally controllable drug release systems, modulating inflammatory networks, restoring immune homeostasis, and mitigating inflammation-associated organ damage.

## 5 Challenges, countermeasures, and future perspectives

### 5.1 Existing challenges

It must be emphasized that the application of AI in sepsis research currently operates within fundamental boundaries: various machine learning and deep learning models perform correlation analyses based on observational data, enabling hypothesis generation, feature mining, and parameter prediction, but cannot independently establish causal regulatory relationships between molecules. All targets identified through AI screening and predicted carrier performance require rigorous *in vivo* and *in vitro* biological validation. At present, a substantial gap remains between research findings and clinical translation, and the potential of AI in precision drug delivery should be viewed rationally, avoiding overextrapolation of expectations.

Although AI-based multi-omics analytical techniques have provided new tools for addressing the core challenges of unclear pathogenic targets and pronounced pathological heterogeneity in sepsis, and nanodelivery systems have offered conceptual and technical references for exploring feasible strategies to overcome the *in vivo* delivery limitations of biomedical and botanical drugs when used alone, and AI-assisted design strategies have provided references for matching nanocarriers, biomedical and botanical drugs with patient pathological subtypes—thereby offering a research framework for constructing novel cross-scale precision therapeutic approaches—the current integrated system combining AI algorithms, nanomaterials, and integrated biomedical and botanical therapies for sepsis remains at the exploratory research and development stage. This system still faces significant bottlenecks across four core dimensions—target mechanism translation, precision nanodelivery, integrated biomedical-botanical synergy, and intelligent design implementation—which severely constrain its transition from theoretical research to clinical application.

Translational bottlenecks in pathogenic targets and pathological subtyping: AI can accomplish sepsis target screening and molecular subtyping based on multi-omics and multimodal data; however, a common challenge persists—“large-scale data screening with insufficient mechanistic validation.” Candidate targets identified by AI based on data correlations cannot readily distinguish true pathogenic driver genes from passive responder genes, lacking causal mechanistic validation. All algorithm-screened candidate targets must undergo systematic *in vivo* and *in vitro* functional validation before they can be applied in subsequent drug matching and carrier targeting design studies. Furthermore, conventional static subtyping models fail to accommodate the dynamic transition characteristics between inflammatory and immunosuppressive subtypes in sepsis, potentially leading to mismatches between therapeutic targets and real-time pathological states. Additionally, organ-specific targets have not yet been precisely matched with the action spectra of biomedical and botanical drugs, resulting in low target utilization and hindering the implementation of molecular subtyping in individualized therapy.

Technical limitations of smart nanodelivery systems: Responsive nanocarriers constructed based on sepsis lesion microenvironment features, while capable of achieving targeted accumulation and controlled drug release, exhibit insufficient individualization adaptability. Conventional carriers with fixed response parameters cannot accommodate the differential microenvironments of different disease subtypes and affected organs; the substantial differences in physicochemical properties between biomedical and botanical drugs make it difficult for conventional nanostructures to achieve stable co-loading and sequential release, compromising the synergistic efficacy of combined biomedical and botanical therapy. Moreover, the dense extracellular matrix at inflammatory lesions impedes nanoparticle penetration, resulting in insufficient drug concentrations at the lesion core and compromising overall therapeutic efficacy.

Implementation bottlenecks of AI-based rational design systems: Although AI can enable full-process intelligent optimization of nanocarriers and individualized decision-making, significant deficiencies exist in practical implementation. Sepsis-related multi-omics data, nanoparticle *in vivo* behavior data, and microenvironmental quantification data are fragmented and lack standardized formats, leading to weak AI model generalizability and low cross-scenario prediction accuracy. Current AI designs are predominantly based on *in vitro* theoretical simulations that cannot fully recapitulate the complex *in vivo* biointeractions and drug release kinetic processes of nanocarriers, resulting in substantial discrepancies between theoretical design and actual therapeutic efficacy. Furthermore, current decision models have not adequately incorporated traditional Chinese medicine syndrome patterns and compound compatibility principles, leaving the integrated intelligent decision-making system for biomedical and botanical convergence incomplete.

Multidisciplinary integration and clinical translation barriers: The cross-scale integration system described herein involves multidisciplinary intersections; however, current research across these fields remains fragmented, lacking systematic validation of the complete “AI subtyping–biomedical/botanical drug compatibility–nanodelivery” chain. Conventional animal models cannot replicate the heterogeneity and dynamic pathological features of clinical sepsis, making it difficult to directly translate laboratory-optimized protocols to clinical patients. Furthermore, AI-customized individualized nanomedicines deviate from traditional standardized drug development, manufacturing, and regulatory review paradigms, and currently lack supporting quality evaluation standards and regulatory frameworks—substantially hindering the clinical translation and implementation of these technologies.

### 5.2 Targeted optimization strategies

To address the aforementioned core bottlenecks, leveraging existing AI algorithms, nanodelivery technologies, and biomedical-botanical research foundations, targeted breakthroughs can be pursued across four key dimensions—mechanism translation, nanotechnological innovation, algorithmic optimization, and clinical translation—to provide direction for refining the AI-driven integrated nanodelivery system for biomedical and botanical drugs.

At the target and subtyping translation level: Establish a closed-loop research framework of “AI screening–experimental validation–target confirmation,” conducting functional validation—including gene intervention and protein blockade—on core pathogenic hub molecules identified by GNN to clarify causal pathogenic mechanisms. Introduce time-series AI algorithms to dynamically capture the patterns of immunological phenotype switching in sepsis, providing references for dynamic patient subtyping and real-time correction of intervention targets. Construct matching databases linking targets with bioactive components and compound formulations of biomedical and botanical drugs, thereby supporting precision medication for patients with different molecular subtypes.

At the nanodelivery optimization level: Leverage AI to quantify microenvironmental parameters across different disease subtypes and affected organs, and develop multi-gradient, multi-logic-gate adaptive responsive nanocarriers to achieve precise matching between carrier performance and lesion pathological features. Construct multi-compartment, core-shell layered nanostructures to enable compartmentalized loading and sequential release of biomedical and botanical drug components, offering design strategies for maximizing the therapeutic value of combined biomedical-botanical approaches. Through AI-iterative optimization of carrier size, surface modification strategies, and penetration properties, enhance the deep lesion infiltration capacity of nanoparticles, providing technical solutions to alleviate the challenge of insufficient drug delivery to the lesion core.

At the AI algorithm upgrade level: Establish multicenter standardized sepsis-specific databases, leveraging federated learning technologies to enable joint modeling across multiple centers, breaking down data silos and enhancing model generalizability. Introduce explainable AI techniques to visualize target regulatory networks, carrier design logic, and drug compatibility rationale, thereby improving the biological plausibility and clinical acceptance of models. Incorporate traditional Chinese medicine syndrome criteria and botanical compound compatibility principles to refine the integrated biomedical-botanical AI decision model, providing references for the dual precision alignment of biomedical molecular subtyping and traditional Chinese medicine pattern differentiation.

At the multidisciplinary integration and clinical translation level: Promote multidisciplinary cross-disciplinary research, conducting full-chain “subtyping–target–drug–carrier” validation in animal models, and refine systematic synergy efficacy evaluation frameworks. Establish clinical translation iteration platforms driven by real-patient multimodal clinical data to facilitate model optimization and carrier iterative upgrading, offering pathways to narrow the gap between theoretical research and clinical application. Drive regulatory system innovation by establishing quality evaluation standards and dedicated review pathways tailored to individualized smart nanomedicines, providing institutional support for the clinical implementation of these technologies.

### 5.3 Future perspectives

Future precision therapy for sepsis will focus on three core directions—cross-scale integration, individualized precision intervention, and synergistic biomedical-botanical convergence—aiming to transcend the limitations of traditional empirical and homogenized therapeutic approaches, while exploring the construction of a novel whole-process data-driven diagnostic and therapeutic research framework. Leveraging spatial multi-omics, single-cell temporal omics, and high-precision AI algorithms, further elucidation of the regulatory mechanisms underlying organ-specific injury in sepsis can be pursued, enabling the discovery of novel pathogenic targets and microenvironmental biomarkers to provide molecular support for nanocarrier targeting modification and precision drug release. Self-adaptive responsive nanomaterials, combined with AI analytical approaches, hold promise for providing foundational theoretical insights into delivery strategies that adapt to the dynamic course of sepsis and achieve matching between carrier release behavior and temporal pathological features. However, significant challenges remain in translating theoretical computational designs into stable and reproducible *in vivo* intervention protocols, with a substantial gap persisting before the development of effective *in vivo* intervention strategies.

In the realm of biomedical-botanical convergence, AI-driven big data mining can elucidate quantitative associations between traditional Chinese medicine syndrome patterns, biomedical molecular subtypes, and pathogenic targets, thereby facilitating the construction of an integrated diagnostic and therapeutic framework encompassing molecular subtyping–pattern differentiation–drug compatibility–nanodelivery. Theoretically, leveraging the differential advantages of biomedical agents in rapid inflammation control and botanical drugs in sustained immunomodulation could provide research directions for developing staged, temporally sequenced synergistic intervention strategies that balance inflammatory suppression and immune restoration. As AI-based design and nanomanufacturing technologies mature, the individualized treatment model of “patient-specific precision therapeutic regimens and customized nanodelivery systems” for sepsis currently remains at the theoretical exploration and early laboratory research stage, with substantial systematic validation work required before preclinical verification can be undertaken. Furthermore, this cross-scale integration framework may offer research references for the precision diagnosis and treatment of other heterogeneous inflammatory conditions, such as severe acute pancreatitis and cytokine release syndrome, providing a potential paradigm for complex critical inflammatory diseases.

## 6 Conclusion

The immunological heterogeneity, dynamic pathological features, and networked pathogenic mechanisms of sepsis have long rendered its precision diagnosis and therapy constrained by three core bottlenecks—unclear targets, inefficient drug delivery, and experience-dependent nanocarrier design—severely limiting clinical translation. This review proposes a conceptual framework for a cross-scale research paradigm encompassing “AI-based mechanistic dissection–nanodelivery-enabled therapy–integration of biomedical and botanical drugs–precision intervention.” The appropriate positioning of AI in sepsis research can be summarized as: a high-throughput screening tool, a hypothesis-generating tool, and a data-integrating tool. Its value lies in mining potential candidate targets from vast datasets, generating experimentally testable scientific hypotheses, and providing computational references for nanocarrier design—yet it cannot directly establish molecular causal mechanisms, nor can it directly inform clinical diagnostic or therapeutic decisions.

At the mechanistic level, AI integrates multi-omics and multimodal data to construct molecular association networks in sepsis, providing computational clues for systematic dissection of pathogenic regulatory mechanisms, technical references for precision molecular subtyping and high-throughput screening of core targets, new tools for alleviating the upstream challenges of unclear targets, subtyping, and mechanisms in traditional research, and a molecular foundation for precision intervention with biomedical and botanical drugs. At the delivery level, nanodelivery systems offer technical references for addressing the shortcomings of biomedical and botanical drugs—mitigating the off-target toxicity and broad-spectrum anti-inflammatory drawbacks of biomedical agents while improving the poor physicochemical properties and low bioavailability of botanical active ingredients. Leveraging the lesion-specific microenvironment of sepsis, these systems provide technical support for targeted drug accumulation and controlled release, constituting a core technological pillar for synergistic biomedical-botanical therapy. At the integration level, AI offers new approaches for transcending the empirical trial-and-error paradigm in nanocarrier development, providing computational bases for data-driven optimization of nanosystems based on target information and microenvironmental parameters, and proposing a conceptual framework for an integrated “target–drug–carrier–subtype” decision model, offering new perspectives for alleviating the blind nature of conventional homogenized therapy.

In summary, AI can bidirectionally link pathogenic mechanistic dissection with nanodelivery regimen optimization, integrating the respective therapeutic advantages of biomedical and botanical drugs to construct a data-driven research framework for individualized precision therapy in sepsis. This framework provides theoretical directions for exploring individualized precision intervention strategies and optimizing inflammatory immune regulatory approaches in sepsis, while also offering insights for the integrated biomedical-botanical research of other heterogeneous severe inflammatory diseases.

## References

[B1] Acosta J. N. Falcone G. J. Rajpurkar P. Topol E. J. (2022). Multimodal biomedical AI. Nat. Medicine 28 (9), 1773–1784. 10.1038/S41591-022-01981-2 36109635

[B2] Alex B. Tomasz R. (2020). Mechanistic understanding from molecular dynamics simulation in pharmaceutical research 1: drug delivery. Front. Mol. Biosci. 7, 604770. 10.3389/FMOLB.2020.604770 PMC7732618 33330633

[B3] Bae H. Ji H. Konstantinov K. Sluyter R. Ariga K. Kim Y. H. (2025). Artificial intelligence-driven nanoarchitectonics for smart targeted drug delivery. Adv. Mater 37 (42), e10239.1–e10239.19. 10.1002/adma.202510239 PMC12548523 40772340

[B4] Baidoo I. Sarbadhikary P. Abrahamse H. George B. P. (2025). Metal-based nanoplatforms for enhancing the biomedical applications of berberine: current progress and future directions. Nanomedicine Lond. Engl. 20 (8), 11–18. 10.1080/17435889.2025.2480051 PMC11999359 40110809

[B5] Brusini R. Varna M. Couvreur P. (2020). Advanced nanomedicines for the treatment of inflammatory diseases. Adv. Drug Deliv. Rev. 157, 161–178. 10.1016/j.addr.2020.07.010 32697950 PMC7369016

[B6] Chen Z. Tang L. Luo L. Luo W. Li Y. Wang X. (2023). Enhancing the treatment of uncontrolled inflammation through the targeted delivery of TPCA-1-loaded nanoparticles. Pharmaceutics 15 (10), 2435. 10.3390/PHARMACEUTICS15102435 PMC10609852 37896195

[B7] Dai L. Sun X. (2026). Stimuli-responsive nanocarriers for precision targeted and controlled antimicrobial drug delivery in drug-resistant infections. Front. Microbiol. 17, 1803769. 10.3389/fmicb.2026.1803769 PMC13303935 42369537

[B8] Dave K. Pazos-García A. Tamarashvili N. Vázquez-Naya J. Munteanu C. R. (2025). Integrated transcriptomic analysis of S100A8/A9 as a key biomarker and therapeutic target in sepsis pathogenesis and AI drug repurposing. Int. J. Mol. Sci. 26 (22), 11186. 10.3390/ijms262211186 PMC12653820 41303672

[B9] Davenport E. E. Burnham L. K. Radhakrishnan J. Humburg P. Hutton P. Mills T. C. (2016). Genomic landscape of the individual host response and outcomes in sepsis: a prospective cohort study. Lancet Respir. Med. 4 (4), 259–271. 10.1016/S2213-2600(16)00046-1 26917434 PMC4820667

[B10] Fangping W. Daphne K. Cesar F. L. D. (2022). Deep generative models for peptide design. Digit. Discov. 1 (3), 195–208. 10.1039/D1DD00024A PMC9189861 35769205

[B11] François B. Lambden S. Fivez T. Gibot S. Derive M. Grouin J. M. (2023). Prospective evaluation of the efficacy, safety, and optimal biomarker enrichment strategy for nangibotide, a TREM-1 inhibitor, in patients with septic shock (ASTONISH): a double-blind, randomised, controlled, phase 2b trial. Lancet Respir. Med. 11 (10), 894–904. 10.1016/S2213-2600(23)00158-3 37269870

[B12] Gaudelet T. Day B. Jamasb A. R. Soman J. Regep C. Liu G. (2021). Utilizing graph machine learning within drug discovery and development. Brief. Bioinform. 22 (6), bbab159. bbab159-1-bbab159-22. 10.1093/bib/bbab159 PMC8574649 34013350

[B13] Garg T. Jain N. Rath G. Goyal A. K. (2015). Nanotechnology-based photodynamic therapy: concepts, advances, and perspectives. Crit. Rev. Ther. Drug Carr. Syst. 32 (5), 389–439. 10.1615/CritRevTherDrugCarrierSyst.2015011645 26559433

[B14] Giamarellos-Bourboulis E. J. Aschenbrenner A. C. Bauer M. Bock C. Calandra T. Gat-Viks I. (2024). The pathophysiology of sepsis and precision-medicine-based immunotherapy. Nat. Immunol. 25 (1), 19–28. 10.1038/S41590-023-01660-5 38168953

[B15] Gokay Y. J E. B. Albert X. Lee A. Bagheri N. Mrksich M. (2019). Exploration of the nanomedicine-design space with high-throughput screening and machine learning. Nat. Biomed. Eng. 3 (4), 318–327. 10.1038/s41551-019-0351-1 PMC6452897 30952978

[B16] Grommes J. Soehnlein O. (2011). Contribution of neutrophils to acute lung injury. Mol. Med. 17 (3/4), 293–307. 10.2119/molmed.2010.00138 21046059 PMC3060975

[B17] He Y. Ye Z. Liu X. Wei Z. Qiu F. Li H. F. (2020). Can machine learning predict drug nanocrystals? J. Control Release 322, 322274–322285. 10.1016/j.jconrel.2020.03.043 32234511

[B18] Hernando G. Can I. Daniel B. D. Pickkers P. Payen D. Hotchkiss J. (2014). A unified theory of sepsis-induced acute kidney injury: inflammation, microcirculatory dysfunction, bioenergetics, and the tubular cell adaptation to injury. Shock (Augusta, Ga.) 41 (1), 3–11. 10.1097/SHK.0000000000000052 24346647 PMC3918942

[B19] Hossan S. M. Gadelrab A. H. Luo M. Guo D. Valenzuela Faccini N. Luo J. (2025). The role of immunomodulatory nano systems in the treatment of sepsis: past, present, and future. Nanomedicine Lond. Engl. 21 (4), 1–23. 10.1080/17435889.2025.2608357 PMC12885438 41452346

[B20] Ismail E. A. Devnarain N. Govender T. Omolo C. A. (2022). Stimuli-responsive and biomimetic delivery systems for sepsis and related complications. J. Control. Release Official J. Control. Release Soc. 352, 3521048–3521070. 10.1016/j.jconrel.2022.11.013 36372385

[B21] Karakike E. Giamarellos-Bourboulis E. J. (2019). Macrophage activation-like syndrome: a distinct entity leading to early death in sepsis. Front. Immunol. 10, 55. 10.3389/fimmu.2019.00055 30766533 PMC6365431

[B22] Komorowski M. Celi L. Badawi O. Gordon A. C. Faisal A. A. (2018). The artificial intelligence clinician learns optimal treatment strategies for sepsis in intensive care. Nat. Med. 24 (11), 1716–1720. 10.1038/s41591-018-0213-5 30349085

[B23] Kwok A. J. Allcock A. Ferreira R. C. Cano-Gamez E. Smee M. Burnham K. L. (2023). Neutrophils and emergency granulopoiesis drive immune suppression and an extreme response endotype during sepsis. Nat. Immunol. 24 (5), 767–779. 10.1038/S41590-023-01490-5 37095375

[B24] Lin S. Ding X. Dang X. Luo D. Cao D. Gong Z. (2026). A multifunctional ICAM1-directed MnO_2_@ZIF8 nanoplatform with Tanshinone IIA synergistically reverses senescence and restores alveolar regeneration in septic lung injury. J. Nanobiotechnology 24 (1), 543. 10.1186/S12951-026-04362-W PMC13251263 42001182

[B25] Marissa M. Suzanne E. Christopher L. Russell A. (2023). AI can help to speed up drug discovery — but only if we give it the right data. Nature 621 (7979), 467–470. 10.1038/D41586-023-02896-9 37726439

[B26] Mason C. Dooley N. Griffiths M. (2016). Acute respiratory distress syndrome. Clin. Med. (Lond). 16 (6), s66–s70. 10.7861/clinmedicine.16-6-s66 27956444 PMC6329572

[B27] Meyer N. J. Prescott H. C. (2024). Sepsis and septic shock. N. Engl. J. Med. 391 (22), 2133–2146. 10.1056/nejmra2403213 39774315

[B28] Mitchell M. J. Billingsley M. M. Haley R. M. Wechsler M. E. Peppas N. A. Langer R. (2021). Engineering precision nanoparticles for drug delivery. Nat. Rev. Drug Discov. 20 (2), 101–124. 10.1038/s41573-020-0090-8 33277608 PMC7717100

[B29] Mogilenko D. A. Shpynov O. Andhey P. S. Arthur L. Swain A. Esaulova E. (2021). Comprehensive profiling of an aging immune system reveals clonal GZMK+ CD8+ T cells as conserved hallmark of inflammaging. Immunity 54 (1), 99–115.e12. 10.1016/j.immuni.2020.11.005 33271118

[B30] Mura S. Nicolas J. Couvreur P. (2013). Stimuli-responsive nanocarriers for drug delivery. Nat. Mater 12 (11), 991–1003. 10.1038/nmat3776 24150417

[B31] Nogales C. Mamdouh Z. M. List M. Kiel C. Casas A. I. Schmidt H. H. H. W. (2022). Network pharmacology: curing causal mechanisms instead of treating symptoms. Trends Pharmacological Sciences 43 (2), 136–150. 10.1016/j.tips.2021.11.004 34895945

[B32] Peer D. Karp J. M. Hong S. Farokhzad O. C. Margalit R. Langer R. (2007). Nanocarriers as an emerging platform for cancer therapy. Nat. Nanotechnol. 2 (12), 751–760. 10.1038/nnano.2007.387 18654426

[B33] Peng Y. Min Yu. Bozhao Li. Zhang S. Cheng J. Wu F. (2025). Advances in nanocarrier-mediated cancer therapy: progress in immunotherapy, chemotherapy, and radiotherapy. Chin. Med. J. 138 (16), 1927–1944. 10.1097/CM9.0000000000003703 PMC12369838 40545573

[B34] Pun F. W. Ozerov V. Ivan Z. (2023). Alex. AI-powered therapeutic target discovery. Trends Pharmacological Sciences 44 (9), 561–572. 10.1016/j.tips.2023.06.010 37479540

[B35] Qiu C. Zhang J. Z. Wu B. Xu C. C. Pang H. H. Tu Q. C. (2023). Advanced application of nanotechnology in active constituents of traditional Chinese medicines. J. Nanobiotechnol 21 (1), 456. 10.1186/s12951-023-02165-x PMC10685519 38017573

[B36] Scicluna P. B. Vught V. A. L. Zwinderman H. A. Wiewel M. A. Davenport E. E. Burnham K. L. (2017). Classification of patients with sepsis according to blood genomic endotype: a prospective cohort study. Lancet Respir. Med. 5 (10), 816–826. 10.1016/S2213-2600(17)30294-1 28864056

[B37] Seymour W. C. Kennedy N. J. Wang S. Chang C. C. H. Elliott C. F. Xu Z. (2019). Derivation, validation, and potential treatment implications of novel clinical phenotypes for sepsis. JAMA 321 (20), 2003–2017. 10.1001/jama.2019.5791 31104070 PMC6537818

[B38] Shen C. Zhang M. Lu M. Chang E. Gao Z. Ban W. (2026). Machine learning empowered formulation design, optimization and characterization of nanoparticulate drug delivery systems: current applications, challenges, and future perspectives. Acta Pharm. Sin. B 16 (2), 665–685. 10.1016/j.apsb.2025.12.011 PMC12891881 41685160

[B39] Singer M. Deutschman S. C. Seymour W. C. Shankar-Hari M. Annane D. Bauer M. (2016). The third international consensus definitions for sepsis and septic shock (sepsis-3). JAMA 315 (8), 801–810. 10.1001/jama.2016.0287 26903338 PMC4968574

[B40] Sun C. Chen J. Bai S. Liao W. Chen J. Guo W. (2025). Recent progress in nano-TCM active ingredient co-delivery systems for inflammation-mediated diseases. Int. J. Nanomedicine 20, 9573–9596. 10.2147/IJN.S526731 PMC12327421 40771757

[B41] Theodoris C. V. Ling X. Anant C. Chaffin M. D. Al Sayed Z. R. Hill M. C. (2023). Transfer learning enables predictions in network biology. Nature 618 (7965), 616–624. 10.1038/S41586-023-06139-9 PMC10949956 37258680

[B42] Timothy E. Sweeney A. Shidham H. R. Khatri P. (2015). A comprehensive time-course-based multicohort analysis of sepsis and sterile inflammation reveals a robust diagnostic gene set. Sci. Transl. Med. 7, 287ra71. 10.1126/scitranslmed.aaa5993 PMC4734362 25972003

[B43] Tom P. D. V. Manu S. Joost W. W. (2021). The immunology of sepsis. Immunity 54 (11), 2450–2464. 10.1016/J.IMMUNI.2021.10.012 34758337

[B44] Topol E. J. (2019). High-performance medicine: the convergence of human and artificial intelligence. Nat. Med. 25 (1), 44–56. 10.1038/s41591-018-0300-7 30617339

[B45] Torres L. K. Pickkers P. van der Poll T. (2022). Sepsis-induced immunosuppression. Annu. Rev. Physiol. 84, 157–181. 10.1146/annurev-physiol-061121-040214 34705481

[B46] Vamathevan J. Clark D. Czodrowski P. Dunham I. Ferran E. Lee G. (2019). Applications of machine learning in drug discovery and development. Nat. Rev. Drug Discov. 18 (6), 463–477. 10.1038/s41573-019-0024-5 30976107 PMC6552674

[B47] Wang W. Ye Z. Gao H. Ouyang D. (2021). Computational pharmaceutics – a new paradigm of drug delivery. J. Control Release 338, 119–136. 10.1016/j.jconrel.2021.08.030 34418520

[B48] Wouters O. J. McKee M. Luyten J. (2020). Estimated research and development investment needed to bring a new medicine to market, 2009-2018. JAMA 323, 844–853. 10.1001/jama.2020.1166 32125404 PMC7054832

[B49] Yang K. Liu B. Bai Y. Chen S. Chen C. Fan J. B. (2026). Emerging nanomedicine strategies for sepsis: immunomodulation and beyond. Res. (Wash D C) 9, 1254. 10.34133/RESEARCH.1254 PMC13100480 42027991

[B50] Yazdani S. Mozaffarian M. Pazuki G. Hadidi N. (2026). Artificial intelligence-assisted design and optimization of stimuli-responsive nanocarriers for smart drug delivery. Mater Today bio. 38, 103153. 10.1016/j.mtbio.2026.103153 PMC13158431 42125257

[B51] Zhang X. M. Liang L. Liu L. Tang M. J. (2021). Graph neural networks and their current applications in bioinformatics. Front. Genet. 12, 690049. 10.3389/fgene.2021.690049 PMC8360394 34394185

[B52] Zhong-ying F. Miao Z. Ning J. L. Zhao X. Zhang Y. q. Fang L. (2021). Tanshinone IIA: a review of its anticancer effects. Front. Pharmacol. 11, 611087. 10.3389/FPHAR.2020.611087 PMC7883641 33597880

